# South Asian women’s lived experiences of health care after disclosure of family violence: a qualitative meta-synthesis review

**DOI:** 10.1186/s12889-025-21619-5

**Published:** 2025-02-04

**Authors:** Surriya Baloch, Elizabeth McLindon, Mohajer Hameed, Kelsey Hegarty

**Affiliations:** 1 Department of General Practice and Primary Care, Melbourne Medical School, Victoria, Australia; 2 Department of General Practice and Primary Care, Melbourne Medical School, The Royal Women’s Hospital, Victoria, Australia

**Keywords:** Family violence, Health system, Identification, Response, South Asia

## Abstract

**Introduction:**

Given the high prevalence of family violence (FV) amongst South Asian women, the experiences and expectations of addressing FV within healthcare, is of policy, practice and research interest. Whilst FV is shaped and influenced by various interconnected sociodemographic and cultural factors, it can be addressed in healthcare settings through identification and response.

**Objective:**

To explore South Asian women's lived experiences and expectations about identifying and responding to FV within healthcare.

**Methods:**

This review utilised a systematic methodology; nine databases were searched up to June 2024. A total of 8,217 records were screened by two reviewers independently based on a priori inclusion and exclusion criteria. A thematic analytical approach guided the integration of findings from 14 qualitative studies.

**Results:**

Thematic synthesis of the articles generated three themes (1) *I was afraid to share*, (2) *They just walk away*, (3) *Understand and listen to my pain*. These themes represented the perspectives, feelings, and expectations of both local and migrant South Asian women survivor participants. Cultural factors and social obstacles may prevent South Asian women from seeking and using appropriate support services. Additional barriers may include healthcare providers’ reluctance to address FV with South Asian women because of a lack of cultural knowledge and/or appropriate methods to address FV. South Asian women participants reflected that they want healthcare providers to understand them, acknowledge their discomfort, and provide culturally appropriate strategies and solutions.

**Conclusion:**

It is highly recommended that policymakers and health-care providers continue to be mindful of the social and cultural challenges faced by South Asian women who experience FV.

## Introduction

Family violence (FV) is a worldwide social and public health phenomenon that impacts families from diverse socioeconomic backgrounds, cultures and religions [1]. The United Nations Declaration on the Elimination of Violence against Women defines FV as emotional, physical, or sexual harm committed inside a private domain, usually amongst people who are intimately related [2]. FV often occurs between close family members, primarily between male and female intimate partners, but characteristics vary from culture to culture [3]. FV is one of South Asia's most serious societal and public health challenges, partly owing to cultural and religious standards that appear to normalise violence [4].

The World Health Organization (WHO) reports that 33% of women experience domestic or sexual violence in South Asian [5]. South Asian countries, including, Afghanistan, Bangladesh, Bhutan, India, Nepal, Pakistan, Sri Lanka and the Maldives, are home to diverse cultures, languages, and traditions [6]. South Asians from Pakistan and Bangladesh predominantly practice the religion, Islam [7]. While India, Sri Lanka, Bhutan, and Nepal exhibit a diverse spectrum of religious adherence, encompassing Hinduism, Buddhism, Christianity, and Islam [7]. Although South Asian people originate from a variety of religious and cultural backgrounds, they often find common ground through shared cultural values. Despite their different origins, shared values such as traditional gender roles, the priority of family, and the stigma against divorce, may foster a sense of belonging among individuals [8]. Sexual or physical violence within marriage is reported by 20% of married women aged 15–49 years in the Maldives [9], 53% in Bangladesh, 38% in India, 28% in Nepal and 26% in Pakistan [10]. The prevalence of FV against South Asian migrant women may be even higher, reported by 40 to 60% of South Asian women in the United States (US) [11]. Both South Asian women living in the US and those living in South Asia are more likely to experience FV than women in other countries [12].

Survivors of FV from diverse cultural backgrounds have distinct experiences and may exhibit varying health outcomes, seek help differently, and navigate systems uniquely. Their susceptibility to FV and associated risks can differ significantly [13]. South Asian women who have moved to other parts of the world may continue to have heightened vulnerability to FV because of sociocultural factors such as deeply ingrained patriarchal beliefs and values [14]. Cultural norms, challenges of lower acculturation and social isolation also complicate routine FV identification or screening of South Asian women, underscoring the importance of effective screening [15–18].

Although FV is a worldwide phenomenon, migrants may be particularly at risk. Research indicates that migrant women are at their highest risk of experiencing FV soon after reaching their destination country [19]. Migrant women in Australia often feel socially isolated, with the majority living in rural or regional areas where access to education, employment and public transport is limited and they do not yet speak English or drive a car [20]. As a result, migrant women may be totally dependent on their partners/husbands, which in turn, may increase their vulnerability to FV [21].

While there is substantial evidence of the high prevalence of FV worldwide, it may be difficult to measure FV against South Asian women [22]. Several socio-cultural expressions of FV are prevalent in South Asian communities, including, high levels of secrecy and close relationships within families that may prevent women from revealing abuse and/or lead to self- blame seeded in culturally-specific beliefs such as ‘karma’ and ‘past life actions’ [23]. In South Asian culture, men are generally socialised to be the dominant family member, while women are enculturated to be subordinate. Women achieve social standing by maintaining their marriages and bearing sons to carry on the family name [24]. Most weddings are arranged by families, with the emphasis on caste rather than mutual affection, compatibility or agreement between the couple [16]. South Asian people often prioritise family above all else, including family honor, harmony, interpersonal connectedness which may prevent women’s disclosure of FV [25, 26]. Furthermore, the bride becomes part of the groom's family and is positioned at the bottom of the family chart [5]. No matter where in the world South Asian women live, that culture pervades, and in some cases, migration may result in a more rigid expression of traditional life and rules as a way of maintaining old-country culture within a new country [12].

Attitudes are one of the key contributors towards FV, as they may shape perceptions of acceptable behavior and what is considered healthy relationships within families [27]. Several factors influence individuals' attitudes and interpretations of FV, including culture, norms, gender, socialisation, laws, and social determinants of health and wellbeing [27]. The WHO multi-country study found that women's views about FV differ considerably across countries [28]. Community surveys about violence-supporting attitudes in Australia identify considerable disparity in attitudes between Australians by birth and Australian migrants from non-English speaking countries [29]. Migrants from non-English speaking countries are much less likely to conceptualise violence as including forced sex, slapping and pushing, stalking or harassment [20]. As a result, reducing violence against women will require not just altering individual views but also confronting the wider societal conditions that underpin such ideas [30].

According to research, the clinical context of healthcare is an optimal site for FV identification and response by healthcare providers (HCPs). International evidence indicated that FV survivors account for 38 to 59% of women who visit healthcare providers [31]. Women use medical services more frequently during their reproductive years [32]. A health-system response that identifies and intervenes with women who have experienced FV is considered crucial for two reasons: to prevent future FV and associated health issues and to lessen the health system burden by connecting survivors with community and specialist FV support services [33]. The clinical context is viewed by some HCPs as an optimal site for FV identification and response since there is trust in the patient-provider relationship [34]. Healthcare providers and policymakers are best positioned to collaborate and support survivors via effective intervention in the healthcare system [35].

In recent years, FV screening and training programs have been implemented in many healthcare systems worldwide [5]. A major challenge in identifying survivors of FV is differing perceptions held by women from various cultures, countries, and ethnicities [36]. Insufficient understanding by HCPs of cultural issues and barriers, undermines the effectiveness and efficiency of screening and identification programs. It is crucial to understand how FV is perceived by survivors in diverse communities to design effective, culturally competent interventions [37]. However, to our knowledge, no systematic review has yet been conducted about South Asian women's lived experiences and expectations about FV identification and response in healthcare settings. To fill this gap in the available evidence, this systematic review aims to explore South Asian women's lived experiences and expectations about identifying and responding to FV in the healthcare setting.

## Method

This review utilised a qualitative meta synthesis methodology which involved the following six steps: (1) the research question was articulated and suitable search terms found; (2) the qualitative evidence was searched systematically; (3) inclusion and exclusion criteria was used to screen retrieved studies; (4) data was extracted from included studies using a data extraction form that was developed; (5) the Critical Appraisal Skills Program (CASP, 2018) qualitative checklist was used for assessing study quality [38]; and (6) findings were synthesised. The research question was defined as follows*: What are the experiences and expectations of South Asian women on identification and response to family violence in healthcare settings?*

## Protocol and registration

The protocol manuscript for this systematic review was prepared and registered to PROSPERO (ID = CRD42021249151).

## Search strategy

A comprehensive list of search terms related to inclusion criteria was created to identify relevant articles; nine bibliographic databases were searched including: Web of SCIENCE (ProQuest); MEDLINE (Ovid); EMBASE (Ovid); CINAHL (EBSCO); Global Health (CABI); PsycINFO (OVID); ASSIA (ProQuest); Cochrane Library (Ovid) and the SocINDEX (EBSCO). Platforms such as Ovid, Ebsco, and ProQuest were used in the search. Using key words and subject headings based on the research question, Ovid and Ebsco search terms included: (1) family violence; (2) screening; (3) South Asia/n; and (4) healthcare setting. A keyword search was conducted on ProQuest with no language restriction or study design or date restrictions; the search took place until July 16th, 2022, and was updated 14th June 2024. The search did not identify any non-English language studies. Additional studies were identified by reviewing reference lists of included studies.

## Study selection

**Fig. 1:**
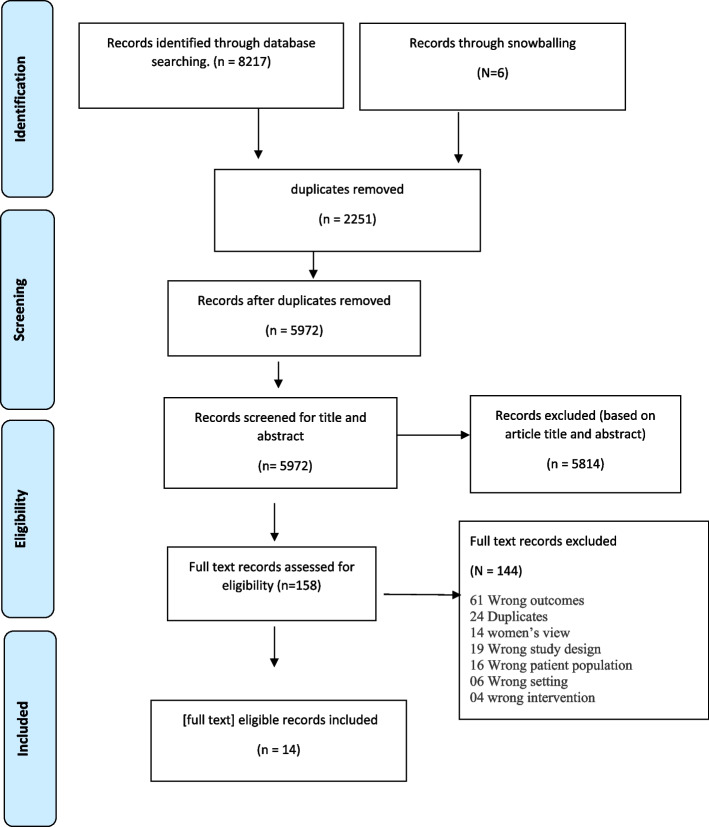
PRISMA Flow Diagram Systematic South Asian women’s views flow diagram showing study selection

The comprehensive database search retrieved 8,217 results, with a further six studies identified from reference lists (see Fig. 1). All retrieved records were imported into Endnote then Covidence, a program to aid screening for systematic reviews [39]. Based on inclusion criteria, two reviewers (SB and MH) screened title and abstracts that: (1) documented South Asian women’s experiences and expectations regarding FV identification and response in healthcare settings; (2) comprised at least 50% South Asian survivor participants; and, (3) used qualitative or mixed methods where qualitative data was collected and analysed separately. We excluded studies that: (1) focused specifically on child or elder abuse; (2) were secondary or grey literature (narrative reviews, editorials, commentaries, case reports, conference proceedings or opinion pieces). Initial screening identified 158 studies as eligible for full-text revision, conducted by SB and MH. Conflict between two reviewers was resolved through continuous consultation with a third reviewer (KH). A total of fourteen studies met the criteria for inclusion in this meta synthesis. Details of the included studies are presented in Table 1**.**

**Table 1:** Characteristics of included studies

Author/year	(country)	Study objective	Qualitative method (analysis)	Setting	Sample size/Demographic
Ahmad-Stout et al., (2018) [43]	United States	The aim of this qualitative study is to describe the experience with IPV among South Asian women living in the United States	Interviews (grounded theory approach)	Three domestic violence agencies in the United States	*N* = 11 (India (*n* = 7) Pakistani (*n* = 3) or Bangladeshi (*n* = 1) (age 24–49 years old) recruited through three domestic violence agencies)
Anitha et al., (2008) [25]	England	To explore their service pathways during and after exit and their experience of health, welfare and legal services; and to inform policy changes based on the research findings	Semi-structured interviews	Social housing	*N* = 30 (South Asian women who had experienced domestic violence)
Decker et al., [52] [52]	India	To describes violence-related coping and help-seeking, and preferences for health care—based intervention, among perinatal women residing in low-income communities in Mumbai, India	Interviews (thematic analysis)	Health center for child immunization or other infant care	*N* = 32 (women who had recently given birth and self-reported recent violence from husbands) (age 15–35 years old)
Janssen et al., [44]	Canada	To hear the views of South Asian women familiar with family violence about ways in which obstetrical care providers could assist them	Workshop (thematic analysis)	Hospital	*N* = 6 (south Asian women survivors of abuse)
Jayasuriya et al. [51]	Sri Lanka	The aims of this study is to identify possible risk/protective factors, and describe the care-seeking behavior of abused women	Interviews (descriptive summaries)	Respondent’s home	*N* = 10 (Family violence survivor ever-married women in the age group of 18 to 49 years)
McCauley et al., [45]	Pakistan, India	To explore what women, consider health and ill health to be, in general, and during and after pregnancy	Focus group discussions (thematic framework analysis)	Healthcare facilities	*N* = 130 (83 women were attending antenatal & 47 postnatal care)
Mukerji et al., [55]	Afghanistan	To addresses the need to understand how domestic violence stigma manifests in women’s lives in such settings, through engaging with the lived experiences of women in Afghanistan	Semi-structured interviews (thematic analysis)	Shelters for survivors of domestic violence provided by NGOs	*N* = 60 (survivors of domestic violence I the age of 18 to 34 years)
Naved et al., [54]	Bangladesh	The study explored at greater length spousal violence against women and collected detailed data on husband’s characteristics, psychological, physical and sexual violence perpetrated by husband, consequences of this violence and coping strategies of a woman	Interviews (thematic analysis)	Household	*N* = 28 (women physically abused by their husbands) (age 15–49 years old)
Poonam et al., (2016) [46]	Nepal	To explore how women who have experienced domestic violence evaluate their antenatal care and their expectations and needs from health centers	Interviews (content analysis)	Two NGOs: Women’s Rehabilitation Center (WOREC) and Community Action Center, Nepal (CAC Nepal)	*N* = 12 (Pregnant women experiencing domestic violence) (age 22–49 years old)
Puri et al., (2005) [47]	United Kingdom and United States	To explores the consequences of multiculturalism-based relativism in health care delivery to battered South Asian immigrant women in England and the United States	Interviews (ethnographic)	Health clinic	*N* = 65 (thirty battered south Asian women US based and thirty-five south Asian women from England
Raj & Silverman, [11]	United States	The purpose of the current study is to assess acquisition of social support as well as social, health and legal services for domestic violence among two community-recruited samples of battered South Asian women	Interviews (grounded theory approach)	Women’s choice location	*N* = 23 (migrant South Asian women in abusive relationship) (age 16–49 years old)
Stokes et al., [48]	Afghanistan	This study explored patterns of abuse and care seeking among women victims of gender-based violence (GBV) in Afghanistan	Interviews (content analysis approach)	Shelter for victims of GBV	*N* = 22 (survivors of GBV) (age 18–26 years old)
Vranda et al., [49]	India	To explore barriers in disclosing IPV to mental health professionals (MHPs) of multidisciplinary team (such as psychiatrists, psychiatric social workers, and clinical psychologists) by women with mental illness experiencing IPV at a tertiary care psychiatric hospital	Interviews (frequency analysis)	Hospital (mental health department)	*N* = 100 (women experiencing abuse) (age 15–49-year-old)
Zakar et al., [50]	Pakistan	This research intends to explore how women cope with SV in the complex and paradoxical setting of Pakistan	Interviews (thematic analysis)	Women’s house and Lady health worker office	*N* = 21 (married women in abusive relation) (age 15–49 years old)

## Data extraction

One reviewer (SB) extracted data from each study into a standardised form which was further reviewed by a second reviewer (MH). Extracted data included: study design, location, mode of data collection, approach to data analysis, characteristics of sample and direct quotes from women accompanied by author interpretation.

## Thematic synthesis

A thematic analysis approach was used to qualitatively identify themes; thematic synthesis was divided into three stages: (1) text coding "line by line”, (2) production of "descriptive themes", and (3) generation of "analytical themes". [40]. The synthesis process was performed by one author (SB) and discussed in detail with the other authors (MH, EM & KH). In line with Thomas and Harden’s methods for thematic synthesis, line by line coding of the result section of each article was conducted. Within qualitative research, this approach provides a systematic and flexible framework for understanding complex phenomena [41]. There are several key stages in the process, including familiarising yourself with the data, generating initial codes, searching and reviewing themes, defining and naming themes, and report writing [40]. After coding, subthemes were re-examined, and each piece of text was compared with others belonging to the same category in order to determine similarities and differences between them. Through thematic analysis, several distinct themes were initially identified from the textual data. However, recognising the interconnectedness of these themes, we embarked on a systematic journey of theme consolidation. By providing clear definitions, specifying boundaries, and identifying relevant sub-themes, we aimed to ensure that our themes were not only descriptive but also conceptually rich. This iterative process enhanced the precision and coherence of our findings, enabling us to draw comprehensive and meaningful conclusions from our qualitative data; contributing to a robust and insightful research narrative. Using an Excel spreadsheet, we combined and refined the themes, subthemes, and participants' verbatim quotes into final broad themes and focused subthemes.

## Quality appraisal

Quality appraisal of all studies meeting the inclusion criteria was conducted by researcher SB using the CASP (Table 2).

**Table 2:** Critical Skill Appraisal Program Questions

Author/year	Clear statement of the aims of the research?	Qualitative methodology appropriate?	Research design appropriate to address the aims of the research?	Recruitment strategy appropriate to the aims of the research?	Data collected in a way that addressed the research issue?	Relationship between researcher and participants been adequately considered?	Ethical issues been taken into consideration?	Data analysis sufficiently rigorous?	Clear statement of finding?	How valuable is the research?	CASP (2018) quality score
Ahmad-Stout et al., (2018) [43]	Yes	Yes	Yes	Yes	Yes	Yes	Unclear	Yes	Yes	Yes	9
Anitha et al., (2008) [25]	Yes	Yes	Yes	Unclear	Yes	Yes	Unclear	Unclear	Yes	Yes	7
Decker et al., [52]	Yes	Yes	Yes	Yes	Yes	Unclear	Yes	Yes	Yes	Yes	9
Janssen et al., [44]	Yes	Yes	Yes	Yes	Yes	Unclear	Yes	Unclear	Yes	Yes	8
Jayasuriya et al. [51]	Yes	Yes	Yes	Yes	Yes	Unclear	Yes	Yes	Yes	Yes	9
McCauley et al., [45]	Yes	Yes	Yes	Yes	Yes	Yes	Yes	Yes	Yes	Yes	10
Mukerji et al., [55]	Yes	Yes	Yes	Yes	Yes	Unclear	Yes	Yes	Yes	Yes	9
Naved et al., [54]	Yes	Yes	Yes	Yes	Yes	Yes	Yes	Yes	Yes	Yes	10
Poonam et al., (2016) [46]	Yes	Yes	Yes	Yes	Yes	Yes	Yes	Yes	Unclear	Yes	9
Puri et al., (2005) [47]	Yes	Yes	Unclear	Yes	Yes	Yes	Unclear	Yes	Yes	Yes	8
Raj & Silverman, [11]	Yes	Yes	Yes	Yes	Yes	Yes	Yes	Yes	Yes	Yes	10
Stokes et al., [48]	Yes	Yes	Unclear	Yes	Yes	Yes	Yes	Yes	Yes	Yes	9
Vranda et al., [49]	Yes	Yes	Yes	Yes	Yes	Yes	Yes	Yes	Yes	Yes	10
Zakar et al., [50]	Yes	Yes	Yes	Yes	Yes	Yes	Yes	Yes	Yes	Yes	10

## Results

### Overview of studies

Fourteen studies published between 2005–2023 were included in this systematic review. Ten of were qualitative [25, 42–50], while four were mixed methods [11, 51–54]. Studies included a total of 539 women experiencing FV, 415 of whom identified as South Asian.

## Study recruitment and demographics

All 14 studies used interviews or focus groups to collect data and applied a thematic analysis approach. Nine studies were from South Asian countries (Afghanistan, Bangladesh, India, Nepal, Pakistan and Sri Lanka), while five studies were conducted in other countries (Canada, USA and UK) with migrant South Asian women (*n* = 135). Participants ranged in age from 15 – 49 years.

## Themes from the review

**Fig. 2:**
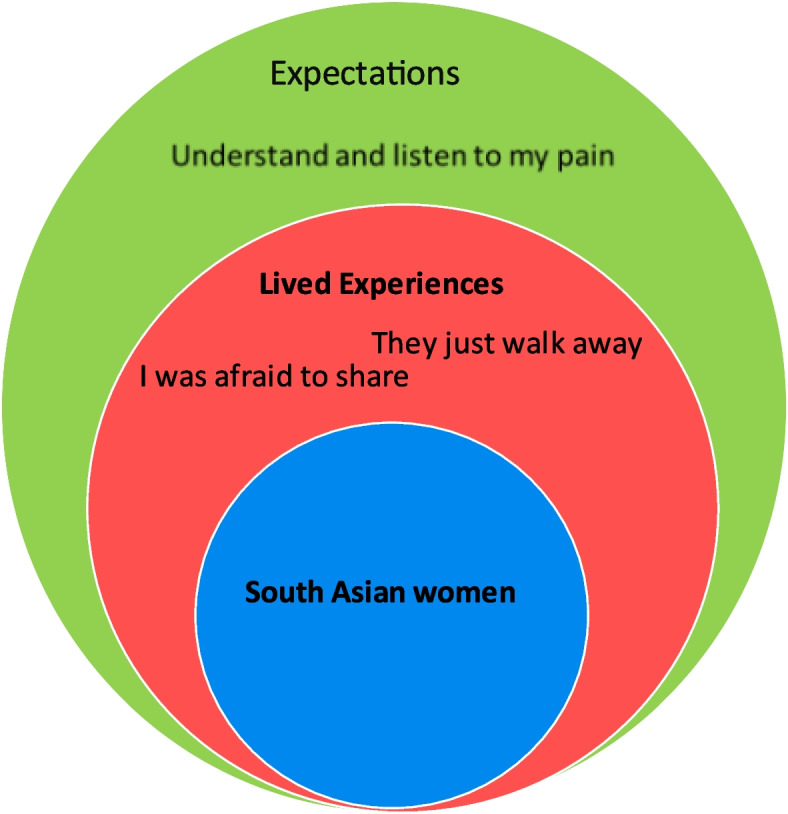
Themes: South Asian women's experiences with disclosures of family violence, and what they expect from health care providers

Thematic synthesis revealed three themes (1) *I was afraid to share,* (2) *They just walk away*, and, (3) *Understand and listen to my pain*. All three themes related to experiences and expectations of FV survivors that prevented help-seeking from HCPs. Issues affecting disclosure included embarrassment as well as family honor fears and concerns by survivors that they would not be helped. Finally, women expected their suffering to be understood by HCPs within a South Asian cultural context Fig. 2.

## Themes

### I was afraid to share

Thirteen studies highlighted considerable ‘silence’ pertaining to sensitive social and health concerns, which appeared to be linked to stigma, family and community influences, loss of privacy owing to continual presence of family, and fear about the consequences of disclosure. [25, 42–54]. Beliefs about violence perpetration being a male partner’s entitlement and fears of jeopardising family honour were the main reasons for being afraid to disclose FV [25, 52, 54]. Women felt embarrassed, guilty and ashamed of revealing FV and taking action was feared to be placing the family unit in danger [51].

*“I told the doctor about how I noticed the bleeding the previous night. I just could not bring myself to tell the doctor about my husband hitting me in my stomach* [during pregnancy]*. After all, my husband is still my husband and I did not want him to be in trouble. I felt very scared and ashamed to tell a stranger about my family problems. The thought of sharing such problems with my doctor was very shameful and embarrassing”* [11], [Interview, United States].

*‘‘If I protest I’ll be marked in the society and then my daughter wouldn’t be able to get married. If I voice my protest the community will blame me for not bearing it in silence. This helplessness is a torture in itself.’’* [54], [Interview, Bangladesh].

Community and family influence and pressure served as powerful barriers to disclosure. Women reported seeing HCPs for the physical and psychological effects of FV, however they did not disclose for fear of being affronted by the community were they to become aware [46]. Women stated that they would conceal the cause of injuries sustained during FV to protect the family from stigma [48].

*“Maybe one and a half years ago* [I noticed the pain]*. My husband threw me against the wall. He hit my head hard on the wall. From that time there is pain all over my head.... The doctor asked me* [if my husband hurt me]*. We told him that I fell down and it happened* [my mum told me to say that I fell by accident]” [48], [Interview, Afghanistan].

*“He* [my husband] *once grabbed my hair and pulled my neck back – I couldn’t move my neck. At the hospital, my mother-in-law told them that I had fallen down the stairs*” [25], [Interview, England].

Lack of HCP privacy and confidentiality was another critical barrier for nondisclosure. Women who met with their HCP when they were not alone could not express themselves freely [46]. The constant presence of their husband and family members during medical consultations muted their ability to discuss their experience and seek help [49].

*“I was scared because of my mother in law, she was there with me, if doctor ask her about it then, I will be caught up by both of my husband and in law…so I didn’t share”* [49], [Interview, India].

*“I washesitant to talk because my elder sonaccompanies me for my hospital visit. I embarrassed to speak infront of him”* [49], [Interview, India].

Women also showed fear of being confined to their houses by their husbands if they came to know about the disclosure of FV, and they were concerned that this would lead to them not being able to visit the hospital for follow up treatment resulting in their abandonment [49].

*“I did not know anyone. I went to the doctor only once because of the swelling* (from abuse)*. But I did not tell them* [HCPs) *what it was. And he* [the perpetrator] *would never take me to the doctor, whatever he did”* [11], [Interview, United states].

Women stated that in South Asian culture, seeking help from formal health institutions was not feasible; they were frightened that to do so could be counterproductive, resulting in increased FV risk [49].

*“I wasactually scared because if he*[husband*) comes to know after confronting with him…… then I am goneforever… (uhh)….hewill beat me like anything after going home”* [49], [interview, India].

*“I told all this* [about the abusive relationship] *to my doctor and told him not to tell my husband. If he comes to know, he will kill me. Doctor promised me he wouldn't tell my husband anything”* [11], [Interview,United states].

Another fear mentioned by migrant South Asian women was being left alone after disclosing FV, with social and financial dependency on the husband necessitating silence [51]. The women who were suffering were terrified that if the authorities arrested their abusive spouse, they would be left in a fragile financial situation, unable to sustain their families {Poonam, 2016 #2}.

*“They* [HCPs] *said it might be a police case, and if I said that I had been beaten by my husband, they would call the police. I would be left alone. I would not have any place to go. I lied and told them that I fell from the ladder while I was carrying a tub… I did not tell anyone”* {Poonam, 2016 #2}, [Interview, Nepal].

In conclusion, South Asian women hid their experiences of FV from HCPs, choosing instead to live with the burden because of shame, abandonment, family dishonor, and stigma fears associated. Furthermore, families of origin and in-laws played an important part in FV and remaining quiet. Women were afraid of reprisal and their lack of autonomy made it much more difficult to take action against assault.

### They just walk away

Eleven studies reported that women had little intention of accessing health services for help because they anticipated judgemental HCPs atittudes. Women reported that HCPs did not usually ask them about FV, and when they did, came across as insensitive and blaming. Only one study reported survivors who had experienced encouraging HCP reactions to their FV disclosure {Poonam, 2016 #2;Ahmad-Stout, 2018 #36;Anitha, 2008 #42;Decker, 2013 #35;Madhani, 2017 #8;McCauley, 2020 #168;Naved, 2006 #23;Raj, 2007 #21;Stokes, 2016 #19;Vranda, 2018 #13;Zakar. R, 2012 #14}. Women reported a lack of empathy in their HCP response, while also perceiving that HCPs could not change their abusive situation {Poonam, 2016 #2}.

*“A woman* [I knew] *disclosed to the HCPs about being abused* [raped] *and that she did not want the baby because the child had no father. The HCPs were afraid that she would run away from the hospital without paying the bill and did not let her go. Nobody sought a solution. That is why I never say anything” *[45] [Interview, Pakistan].

*“I will never tell a health worker especially nurses because they have their own way… they just walk away” {Poonam, 2016 #2},* [Interview, Nepal].

While describing her concern about of contacting health services, one survivor woman said that the doctor did not assist with referral to a FV service [11]. Women believed that the HCPs did not adequately follow them up or advise them about support services {Raj, 2007 #21;Poonam, 2016 #2}. Women highlighted that HCPs often failed to understand the root cause of their suffering. Women stated that while looking at their presenting issues and health symptoms, the HCP should consider the underlying reason [43]. Women have reported that their interactions with the healthcare system frequently result in stigmatising experiences, where healthcare providers either dismiss women's accounts or remain silent about the violence. [55].

*“I haven't received any help … I meet them* [the social workers] *every week. But I'm not getting any help from them. They just come and talk to me.” *[11]*,* [Interview, United states].

*“I tried to commit suicide twice. Once I took poison and my mother-in-law and sister-in-law took me to [name of hospital]. The hospital didn’t investigate at all. I want to request hospitals to involve the police and investigate when a woman tries to poison herself. Both times I was taken to the hospital and both times woke up athome without anyone asking me why I did it.” *[55] [Interview, Afghanistan].

Women in the studies thought it would be ineffective to disclose FV to HCPs. As women had no one to safely share their expereince with, they described FV as a situation for which no practical choices exist [52]. Despite barriers, women struggled to approach services to find the help they needed to leave the violent relationship, often repeatedly [25]. One women managed to talk tp a HCP about sexual violence, asking for help to stop sexual violence against her but she was disoppointed with the HCP’s judgemental and insensitive response {Poonam, 2016 #2}:

*“Then he told me that I should talk to my husband and it was my responsibility to convince my husband. He used words like maybe you also need sex. I felt very bad because he talked to me like that. He was a male doctor” {Poonam, 2016 #2},* [Interview, Nepal].

*“I couldn’t talk about the sexual abuse as I was too embarrassed. … I started getting headaches and taking paracetamol. Eventually I told the doctor a bit about what was happening at home. He said, ‘Don’t think about it.’ But I say, how can I not think about it?” *[25]*,* [Interview, England].

Findings of this synthesis show that despite a number of obstacles, South Asian survivor women had made efforts to access healthcare services to find support. However, their experiences of services remained highly varied and inconsistent [25]. In contrast to the studies canavased so far in this theme, one study found that many women reported that mental health providers responded empathetically to their disclosure of violence and they were provided support. Information about support services and legal options was provided to them to help them protect themselves and their children from further abuse. [49]. They made comments such as:

*“She listened patiently to what I said,” “she was supportive and gentle,” “he allowed me to speak,” “told not to hide these issues and encouraged me to seek help from my parents/friends,”* and *“she instilled confidence in me” *[49]*,* [Interview, India].

### Understand and listen to my pain

Six studies reported how South Asian women shared what they wished from HCPs, including an opportunity to discuss their experience, get support and help {Poonam, 2016 #2;Ahmad-Stout, 2018 #36;McCauley, 2020 #168;Vranda, 2018 #13;Janssen, 2009 #167;Puri, 2005 #22}. Across the studies, South Asian women who experienced FV expressed support for screening, as long as it was private and confidential, and HCPs were nonjudgmental in their provision of safety and support [49].

*“I told you* [interviewer] *because you seem to understand my pain’. Women said that empathy, politeness, love, and respect would motivate them to share their experience of DV. ‘… They* [HCPs] *should ask about what had happened to us, how we got to the hospital, who abused us’, said one of the women” {Poonam, 2016 #2}, [Interview, Nepal].*

Women suggested that HCPs should inquire about FV in culturally appropriate ways, inquiring about mental and social illness, including FV, during the routine consultation [45]. Further, they reported that routine enquiry about FV could reduce the stigma and shame associated with it [44]. According to women, speaking with their HCPs about their experience of violence would be beneficial, they might be able to provide general care and support to reduce their pain and stress [45].

*‘If one has some grief, sharing with someone lessens the grief, though it may not solve the problem, but releases stress”* [45], [Interview, Pakistan].

*“They should use questions in a way that are focused and clear”* [45], (FG, Pakistan).

*“Do you have any tension? How is your home environment? How is your husband and your family- in-laws behaviour with you?”* [45], [Interview, Pakistan].

Participants in two studies said that they were mostly just assigned to South Asian HCPs because of their shared cultural background and an assumption that South Asian HCPs may understand and respond to FV survivors more appropriately [45, 47]. However, South Asian women did not mention that they had received more support and care from HCPs who shared their cultural background [47].

*“Look, just because I’m Muslim and my GP might be Muslim, it doesn’t mean he or she is going to understand and help me! For all I know, they might give me a hard time for wanting to leave or saying anything at all about my husband. It’s a fairly big jump, isn’t it, to say that Asians will always support and understand Asians, right?”* [47]*,* [Interview, United Kingdom].

Women suggested that it would be useful to talk to their HCP if they were under stress and unwell, as the HCP might be able to provide some support and guidance. Most women wanted HCPs to inquire about their social health and wellbeing during routine comsultation, however, women’s expereince was of a lack of enquiry from healthcare practitioners regarding emotional health [45].

*‘They ask in detail about me, about my diet and medicines, they take a complete history and ask about all my previous deliveries, but they never ask about my family environment or mental health”* [45]*,* [Interview, Pakistan].

South Asian women expected healthcare providers to be sympathetic to survivors of family violence, inquire about their experiences, ensure confidentiality and privacy, and refer them to appropriate services. Supporting and identifying these women could lead to better services for them.

## Discussion

This systematic qualitative meta-synthesis identified fourteen studies which explored South Asian women’s experiences and expectations of healthcare providers after disclosure of FV. In my analysis, I have combined South Asian women into a single group despite their diverse ethnicities and regional backgrounds. This approach is based on the understanding that, while there are notable differences among South Asian subgroups, they often share core cultural practices, values, and social norms. For example, South Asian cultures broadly emphasize family structures, hierarchical relationships, gender roles, and community cohesion [56]. These shared values influence experiences, particularly in terms of family dynamics, healthcare decision-making, and attitudes about sensitive topics such as family violence [16]. Further, cultural practices such as arranged marriage, deference to elders, and modest behavior, are prevalent across many South Asian communities, regardless of specific ethnicity or region [57]. The overarching pattern of findings indicated three broad themes: *I was afraid to share; They just walk away;* and, *Understand and listen to my pain.* Firstly, this review found that South Asian women were reluctant to disclose FV due to various factors, including family honor, shame and stigma, fear about the consequences of disclosure, financial dependence on the perpetrator and the influence of family. The findings of this meta-synthesis suggest that family and broader community attitudes towards South Asian women who attempt to escape abuse and violence may discourage disclosure, compounding silence about FV [11, 44, 49]. A South Asian woman's ability to remain in an abusive relationship is associated with her sense of family honor in community [25, 54]. As a result of internalising social values, women may be afraid of sacrificing family honor, deepening their suffering [54].

These findings are consistent with literature about South Asian women who conceive of their role as silent and obedient partners responsible for family honor protection and management of the home [5]. However, previous reviews have shown that the impact on women may vary, influenced by women’s socio-cultural background, race, family background and religious affiliation [38]. Consistent with previous studies, this meta-synthesis found that the experience of survivors living in South Asia was similar to that of South Asian migrants living in other parts of the world [48, 54]. Family violence affects women globally, although there are culturally specific issues, such as culturally mediated shame and fear, especially in terms of seeking help and support from HCP [11, 44, 48, 49, 58]. A woman's inability to leave a violent relationship may be attributed to family and societal pressures and stigmas through which women are forced to keep the family together at all costs [11, 42, 49]. Our findings are supported by literature which demonstrates that cultural stigma is likely the predominant barrier to FV disclosure, with fear of humiliation and community disgrace playing a pivotal role in lack of disclosure and help-seeking [59].

Studies in this meta-syntheses stressed the importance of privacy and confidentiality for women visiting healthcare providers {Poonam, 2016 #2;Vranda, 2018 #13;Janssen, 2009 #167}. South Asian women were concerned about being assessed by members of their own ethnic group, as well as personal information being leaked via interpreters from within their local community [44, 47, 49]. Broadly, South Asian women feared being judged by others and were cautious about sharing personal information with them [5]. Mostly women are accompanied by a family member during health consultations, posing a major barrier to FV disclosure [42, 49]. A similar view was expressed in another review of migrant South Asian women and help seeking which found HCPs reluctant to report abuse if they suspected the perpetrator was present [5]. Although South Asian women and migrant South Asian women experienced and interpreted FV in similar ways, migrant women may face a heavier burden of harm and additional barriers to help-seeking due to challenges specific to immigration, including legal status and financial dependence on perpetrator [11, 42, 44]. Evidence shows that among ethnic minority populations in the UK, South Asian women reported that they could not seek help due to dependency on their husbands and immigration status, they had no access to refuge, appropriate housing or welfare payments [60].

Findings from the theme, *they just walk away,* demonstrated that women were receiving sub-optimal care*.* For the sake of her family's respect, a South Asian woman may initially stay in the relationship and home, tolerating and correcting extended family oppression. However, over time, health issues (depression, anxiety, headaches, gastrointestinal symptoms), worsen, often necessitating medical care [5]. South Asian women who have been abused and sought help may require both medical and emotional support [43]. Evidence shows that despite the extent and harm of FV, healthcare responses in South Asian countries have been limited [33] Healthcare is essential for survivor identification and response and for referral to multi sectorial approach [11]. However, family violence is not considered the responsibility of healthcare providers, who often do not know how to identify survivors or what to do if a woman discloses [33]. Further, this meta-synthesis revealed that South Asian women who had disclosed were frequently met with HCP insensitivity and blame {Poonam, 2016 #2}. Consistent with previous literature, HCPs commonly ignored the underlying reason for suspicious injuries, fuelled by negative attitudes towards women [5].

Despite these barriers, women participants in this review wanted to disclose their experience of violence to their HCPs, wanting a healthcare provider to understand, recognise their pain, and support pathways to safety [49]. Our findings reinforce those studies that have shown the experience of women who have repeatedly sought help from the healthcare system to no avail [42]. Women wanted HCPs to routinely enquire about FV according to their culture, religion and context; with a recovery-oriented response {Poonam, 2016 #2}. Research shows that eliminating the stigma about FV may be key to HCPs comfortably asking about the issue, while signaling to women that they can disclose without fear or shame [5]. Women emphasised that when they were asked about FV, keeping an understanding of their culture, religion, and context can be a positive experience if done in a standard routine matter [45]. Our findings are supported by studies that emphasise the need for culturally specific healthcare responses that may break down some of the shame and stigma felt by South Asian survivors [4]. In this review same-gender care providers who understood culture and spoke a local language were preferred by migrant South Asian women in Western countries [43, 47]. This review found that women wanted to be heard, understood, and supported to leave traumatic situations of FV [43, 49]. Similarly, Jejeebhoy (2014) noted that providers who interact with women should be proactive and sensitive in identifying FV and supporting survivors to share their story of abuse. The O’Doherty et al. (2014) Cochrane review noted that implementing screening procedures within the healthcare system could enhance the detection of women who have experienced violence [61]. Furthermore, this screening recommendation operates on the assumption that the identification process will lead to suitable interventions and support, thereby reducing future incidences of violence and mitigating detrimental health consequences [62].

### Implications

This review highhighted South Asian women’s lived experience of FV identification and response from HCPs. This meta-synthesis recognised numerous factors occuring at multiple levels (e.g. family, community, society) associated with South Asian women’s healthcare help-seeking. Amogst many inter-related barriers to identification, was feeling unable to disclose due to stigma, fear about the reactions of family and community, privacy and fear of impacts of disclosure. Further, HCP’s who lacked empathy or displayed a judegemental atittude towards women’s disclosure, were a barrier to women disclosing FV. However, healthcare is likely to provide opprotunites to identify and aid South Asian women who are survivors of FV. Women participants were keen to be asked questions about FV and receive simple suggestions and support. These findings highlight the significance of providing culturally appropriate FV screening and psychosocial support for women in healthcare settings.

These findings may contribute towards the co-design and development of culturally sensitive programs addressing family violence. It is crucial that healthcare providers are aware that many South Asian women who experience FV may not wish to leave their families, and their choices ought to be respected [11]. Culturally relevant services and interventions that support South Asian women may consider factors such as disgrace and stigma related to FV throughout the processes of identification and response [63]. Sociocultural risk factors associated with South Asian women have not been adequately incorporated into holistic understandings of their experiences. Researchers and practitioners can use these gaps to build future knowledge and address important implications for research and practice.

### Strengths and Limitations

This is a comprehensive systematic review of socio-cultural risk factors amongst south Asian women, which may influence FV identification and response in healthcare system. The included qualitative studies utilised a variety of theoretical frameworks and approaches which considerably adds to the credibility and validity of these findings. The inclusion of publications from South Asian nations as well as from the United Kingdom, the United States, and Canada, enhances the generalisability of the findings. Although the findings may not apply and/or relate to other ethnic minority populations. This review has several limitations such as the inclusion of a relatively small number of wholly qualitative studies. Indeed, the limited number of studies available on this topic is a constraint that we acknowledge. While our findings provide valuable insights within the scope of the existing literature, we understand that caution should be exercised when attempting to generalise our results beyond this limited dataset. It's essential to emphasise that our study serves as a valuable contribution by shedding light on a relatively unexplored area, and we hope it will inspire further research and data collection efforts to bolster the evidence base. Although a comprehensive search strategy was used with PRISMA guidelines, it is possible that other studies that match the inclusion criteria may have been overlooked.

## Conclusion

This review utilised a systematic methodology to synthesise the findings from qualitative studies which explored the needs and lived experiences of FV survivors from South Asia (Table 3). This review highlighted that South Asian women have unique religious, cultural, and physical demands that may differ from women from other ethnic minority groups [63]. South Asian women reported that the stereotyped attitude of healthcare was inappropriate and disturbing, and it had a detrimental influence on their help-seeking behaviours. Despite these challenges, women want to tell their HCPs about their experiences with family violence. There is a need for them to find a healthcare provider who can understand their problems, consider their sorrow, and provide suitable and appropriate solutions. Our review demonstrated gaps within the literature on FV and South Asian women with more research required to provide better care for diverse migrant survivors of family violence. In summary, the papers evaluated in this review provide useful recommendations that may aid policymakers and service providers to better provide assistance to women survivors from South Asian cultural backgrounds. Our study underscores the pressing need for further research in this field, as it has illuminated important avenues of inquiry and highlighted complexities warranting deeper exploration to enhance our understanding and inform evidence-based solutions. Specifically, future research could focus on the cultural adaptation and acceptability of screening tools among diverse populations. Further, longitudinal studies are needed on the impact of early intervention, and the effectiveness of training programs for healthcare providers in culturally sensitive screening practices. Research in this area would address the identified gaps and contribute to more effective, culturally responsive strategies to better identify and responded to survivors of family violence through antenatal screening. Current international research often focuses on women from South Asian ethnicities for analysis, grouping them together under the broad label of "South Asian." However, it is important to recognise that this term encompasses a wide range of cultures, languages, and social contexts. As a result, the diverse experiences and identities of individuals within this group may be overlooked, which could lead to generalised conclusions that do not accurately reflect their unique backgrounds. Understanding the specific nuances among South Asian communities is essential for a more comprehensive and meaningful analysis of their experiences and challenges. Table 3 shows summery of critical findings of this study. A summary of the meta-synthesis' implications is presented in Table 4 (Table 4).

**Table 3:** Summary of critical findings

According to South Asian women, the main reason why they did not disclose family violence to health care providers (HCPs) was: *I was afraid to disclose*
• Based on the data, most women chose to conceal their abuse from healthcare practitioners for fear of social stigma and shame
• Women were scared of retaliation if their husband learned about the abuse allegations
• The presence of husbands and family members prevented women from disclosing abuse during medical consultations
• Migrant South Asian women also feared being left alone after disclosing FV, since they depended on their husbands for economic and social support
Additionally, South Asian survivors have made efforts to access health care services to find support, but they feel that HCPs are insensitive and discriminatory:* They just walk away*
• According to women, they did not disclose their FV experiences because of lack of empathy from the HCPs
• Despite of managing to talk to the HCP and asking for help, women reported being disappointed with the HCP's judgmental and insensitive response
• Findings revealed that women presented with physical symptoms, but their health care providers did not understand the underlying cause of their distress
When it comes to what South Asian women wanted from HCPs, they wanted opportunities to discuss their experiences with them and to get support and help from them*:* *Understand and listen to my pain*
• South Asian women suggested that health care practitioners may inquire about FV during routine consultations in culturally appropriate ways, asking about mental health and social ill-health including FV
• Health care providers are expected to be sympathetic to the experiences of South Asian women who have suffered from family violence, inquire about their experiences, ensure their privacy and confidentiality, and suggest suitable services

**Table 4:** Implications for practice, policy and research

Practice
•Healthcare organizations must prioritize cultural competency training and integrate it into their staff development programs. This includes understanding cultural nuances, communication styles, and taboos that may impact disclosure
•Routine screening for family violence, with a focus on cultural sensitivity, should be incorporated into the standard practices of healthcare providers. This approach can help identify cases early and provide timely support
•Implementing trauma-informed care principles is essential, Healthcare providers should recognize the potential trauma experienced by South Asian women and create a safe and non-retraumatizing environment
**Policy**
•Engage in community advocacy and awareness campaigns to empower South Asian women to seek help and reduce stigma associated with family violence
•Healthcare organizations should engage in policy advocacy to address gaps in legislation and healthcare policies related to family violence, ensuring the protection and support of survivors
•Healthcare organizations should establish mechanisms for continuous monitoring and evaluation of their family violence identification and response programs to assess their effectiveness and make improvements
**Research**
•Encourage research initiatives and data collection efforts that focus on family violence within South Asian communities. This research can inform tailored interventions and improve understanding
•It is imperative that future studies focus on the development and implementation of health systems that can provide culturally competent interventions to South Asian women

## Data Availability

This Study is a systematic review and meta-synthesis based on data extracted from previously published studies. All data supporting this study's findings are available in the original published articles, which are cited in the manuscript. No new data were generated or analyzed during this this study.
